# Radiologic-pathologic correlation of prostatic cancer extracapsular extension (ECE)

**DOI:** 10.1186/s13244-023-01428-3

**Published:** 2023-05-16

**Authors:** Adalgisa Guerra, Beatriz Flor-de-Lima, Gonçalo Freire, Ana Lopes, João Cassis

**Affiliations:** 1 Department of Radiology, Hospital da Luz Lisboa, Avenida Lusíada 100, 1500-650 Lisbon, Portugal; 2 Faculdade de Ciências Médicas, NOVA Medical School, Lisbon, Portugal; 3 Radiology Department, Centro Hospitalar Tondela-Viseu, Viseu, Portugal; 4 Radiology Department, Hospital da CUF, Lisbon, Portugal; 5 Pathology Department, Hospital da Luz Lisboa, Lisbon, Portugal

**Keywords:** Prostate, Prostatic cancer, Extracapsular extension, Magnetic resonance imaging, Radiologic-pathologic correlation

## Abstract

**Abstract:**

Recent advancements on nerve-sparing robotic prostatectomy allow fewer side effects such as urinary incontinence and sexual dysfunction. To perform such techniques, it is essential for the surgeon to know if the neurovascular bundle is involved. Despite being the gold-standard imaging method for Prostate Cancer (PCa) staging, Magnetic Resonance Imaging (MRI) lacks high specificity for detecting extracapsular extension (ECE). Therefore, it is essential to understand the pathologic aspects of ECE to better evaluate the MRI findings of PCa. We reviewed the normal MRI appearance of the prostate gland and the periprostatic space and correlated them to prostatectomy specimens. The different findings of ECE and neurovascular bundle invasion are exemplified with images of both MRI and histologic specimens.

**Graphical abstract:**

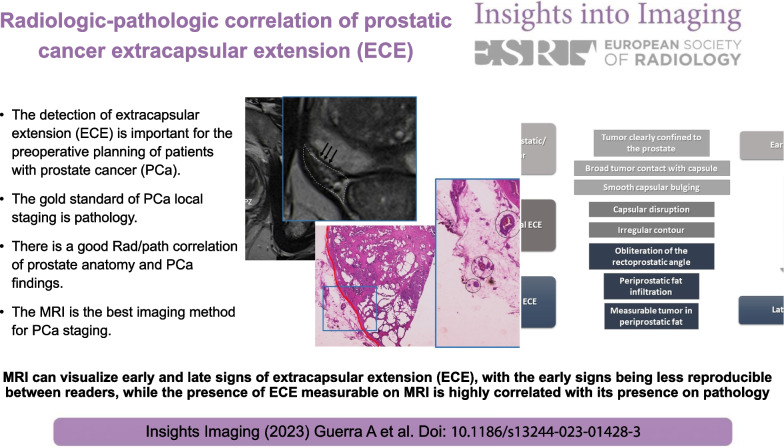

## Abstract (artclehighlights)

**Critical relevance statement:**

MRI can visualize early and late signs of extracapsular extension (ECE), with the early signs being less reproducible between readers, while the presence of ECE measurable on MRI is highly correlated with its presence on pathology.

**Key points:**

- Detection of extracapsular extension (ECE) is important for the preoperative planning of patients with prostate cancer (PCa).
- The gold standard of PCa local staging is pathology.
- There is a good Rad/Path correlation of prostate anatomy and PCa findings.
- The MRI is the best imaging method for PCa staging.

## Background

Extracapsular extension (ECE) detection is crucial for the preoperative management in patients with prostate cancer (PCa). MRI is the gold-standard imaging method for locoregional staging of PCa [1]. The classical features to detect ECE on MRI are suspicious for microscopic ECE, but the sensitivity is heterogeneous among different studies [2].

Recent advancements on surgical technique allow nerve-sparing robotic prostatectomy with likely negative surgical margins in patients with organ-confined PCa. This minimally invasive technique averts side effects such as urinary incontinence and sexual dysfunction. Therefore, it is essential for the surgeon to know if the neurovascular bundle is involved.

Therefore, our goals are to review the appearance of the normal prostate and of the neurovascular bundles on MRI, comparing it to prostatectomy specimens. Additionally, it is our aim to correlate the MRI images with the histologic findings of prostatectomy specimens in patients with PCa treated with Robotic-Assisted Radical Prostatectomy (RARP). The present review appears to illustrate some educational cases that were selected from a pool of patients analyzed in a previous observational study conducted by the first author. The previous study analyzed the MRI features of 169 patients who underwent RARP at the first author´s institution and correlated those features with the presence or absence of ECE on pathology specimens [3].

## Imaging findings

### MRI protocol for PCa staging

Recommended use of MRI in prostate cancer consists of multi-parametric MRI (mpMRI), which includes a combination of T2-weighted images (T2WI) and functional MRI techniques such as diffusion weighted imaging (DWI) and dynamic contrast-enhanced (DCE) [4]. For evaluating minimal ECE, preferably, the examination should be done at 3 T, but 1,5 T equipment is also recommended, including high spatial resolution T2WI in axial, coronal and sagittal planes covering the entire prostate. The functional sequences should be done mainly for detection and localization of the tumor and also for assessment of periprostatic space and seminal vesicle invasion [5].

### Normal anatomy of the prostate and periprostatic space

The peripheral zone (PZ) makes up most of the prostatic gland in young adults. It has a characteristic high-signal intensity on T2WI (greater than adjacent fat), with a typical cup-like shape [6].

**Fig. 1:**
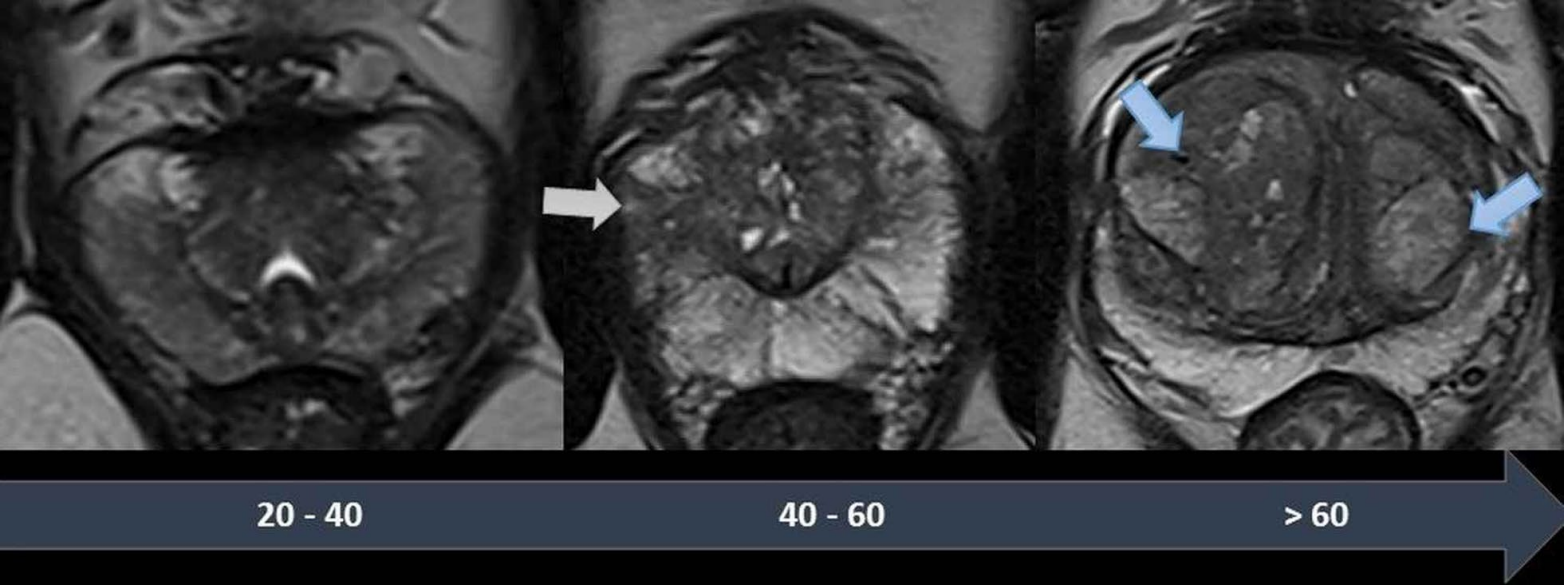
Timeline showing the different physiologic features of the normal prostate on T2-weighted MR images. Younger patients have a dominant PZ, which is homogeneous and shows low signal-intensity. In middle-aged men, non-nodular T2-hypointense bands can appear in the PZ, in relation with prostatitis (grey arrow) and the TZ becomes larger. In older patients, the TZ becomes dominant, with multiple well-delineated nodules, corresponding to benign prostatic hyperplasia (blue arrows). Reprinted with permission from Flor-de-Lima B, Freire G, Lopes A, et al. (2022) Radiologic-pathologic correlation of prostatic cancer extra-capsular extension (ECE) (C-18805) EPOS™ poster presented at ECR 2022

The central zone (CZ) makes up about 1/4 of the prostatic gland volume. Both central and transition zones have signal intensity lower than the peripheral zone on T2WI. The transition zone (TZ) of prostate gland typically enlarges with age due to benign prostatic hyperplasia (BPH), which manifests as varying number of typical circumscribed hypointense or heterogeneous nodules on T2WI. These could manifest as multiple, similar, scattered restricted diffusion nodules throughout TZ. Sometimes these nodules are incomplete or almost completely encapsulate nodules on T2WI and are also assigned as atypical nodules of BPH. In this case, the high DWI could be important to differentiate them from indeterminate nodules with high diffusion restriction [7]. When the CZ individualized from TZ appears as a symmetric band or “dumbbell-shaped” appearance, of tissue between the PZ and TZ at the base of the prostate, below seminal vesicles to the verumontanum and surrounding ejaculatory ducts and exhibits decreased signal intensity on T2WI and apparent diffusion coefficient (ADC) [8]. Prostatic morphology changes with aging, and those changes are shown on Fig. 1.

The prostate is not demarcated by a true capsule, but rather by a pseudocapsule which consists of fibromuscular tissue. It is divided into three fascial layers, which are sparser anteriorly and in the apex. The pseudocapsule is a thin line that contours the prostate gland, with low signal intensity on T2-WI. The anterior fibromuscular stroma also has low signal intensity in both T2 and T1-weighted images [2, 6].

The neurovascular bundles (NVB) are a crucial landmark for surgery, and several anatomic variants regarding its location are known. Notwithstanding, they are commonly located posterior and laterally at 5 and 7 o’clock, branching to both apex and base and at a distance of about 3 mm from the capsule.

**Fig. 2:**
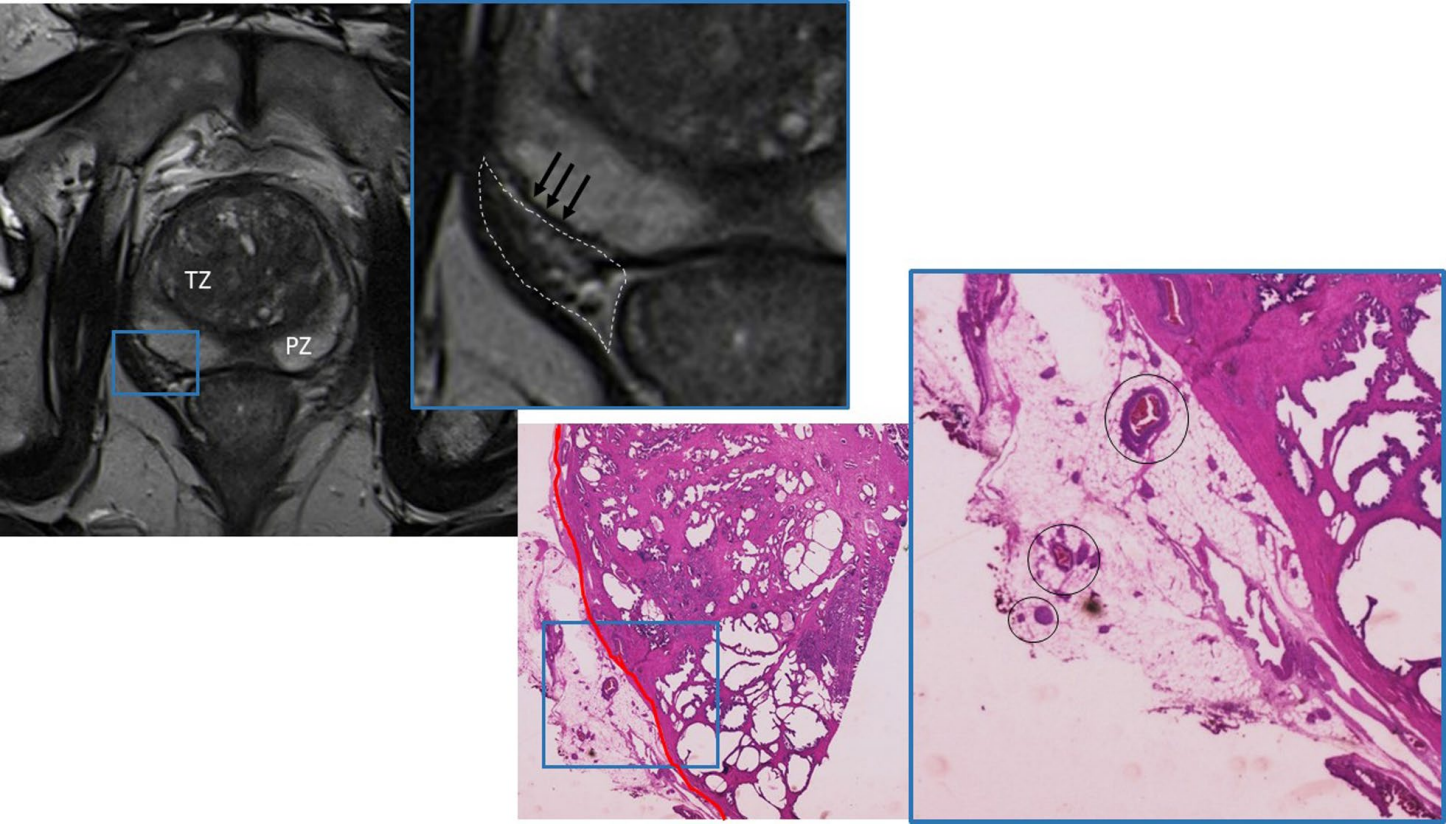
Radio-pathological correlation of normal prostatic anatomy. T2-weighted image MR image demonstrating the TZ with low signal intensity, surrounded by the PZ with high signal intensity. The prostate is limited by a pseudocapsule, seen as a thin low signal intensity rim (black arrows). Beyond its boundaries lies the periprostatic space (dashed line), containing fat (depicted as high-signal intensity), nerves, and vessels (tubular structures with low signal intensity). The correspondent histological specimen illustrates the prostatic capsule (red line) and the periprostatic space, containing fat, nerves, and vessels (Hematoxylin and Eosin (H&E)). Reprinted with permission from Flor-de-Lima B, Freire G, Lopes A, et al. (2022) Radiologic-pathologic correlation of prostatic cancer extra-capsular extension (ECE) (C-18805) EPOS™ poster presented at ECR 2022

The periprostatic tissue comprises adipose tissue, small blood vessels, lymphatics, the periprostatic fascia, the posterior prostatic fascia, and seminal vesicles fascia (Denonvillers) and other structural ligaments that hold the prostate (Fig. 2) [9].

### The typical appearance of prostate cancer on MRI

Typically, PCa presents as a hypointense lesion on T2-weighted imaging and reduced values on apparent diffusion coefficient (ADC) map and as a high signal lesion on high-b-value (> 1400 s/mm^2^). On dynamic contrast-enhanced MRI (DCE-MRI), it normally appears as an early focal enhancement [6].

On multiparametric MRI, the Prostate Imaging Reporting and Data System (PI-RADS) is an assessment category of five different categories according to the lesion's risk of malignancy, firstly published in 2012 [5]. The last PI-RADS version (v2.1) consists of a combination of imaging findings on T2-weighted, DWI and DCE images, and each lesion is graded between 1 and 5. PI-RADS 1 and 2 indicate probably benign lesions, PI-RADS 3 indicates a lesion of unknown significance, and PI-RADS 4 and 5 indicate lesions with a high or very high probability of malignancy that should undergo histological evaluation. The category PI-RADS 5 also includes definitive extraprostatic extension or invasive behavior of the lesion [4].

### Prostate cancer staging on MRI

Staging is determining in the treatment choice in patients with PCa, as different possibilities are available whether the disease is prostate-confined, locally advanced or distant disseminated [10].

Patients with locally advanced disease have a worse prognosis than those with prostate-confined tumors (pathological T1–T2). The most important aspects regarding the T-staging are ECE (pathological T3a tumors), seminal vesicles invasion (T3b tumors), and invasion of adjacent structures others than seminal vesicles (T4 tumors) [2].

**Fig. 3:**
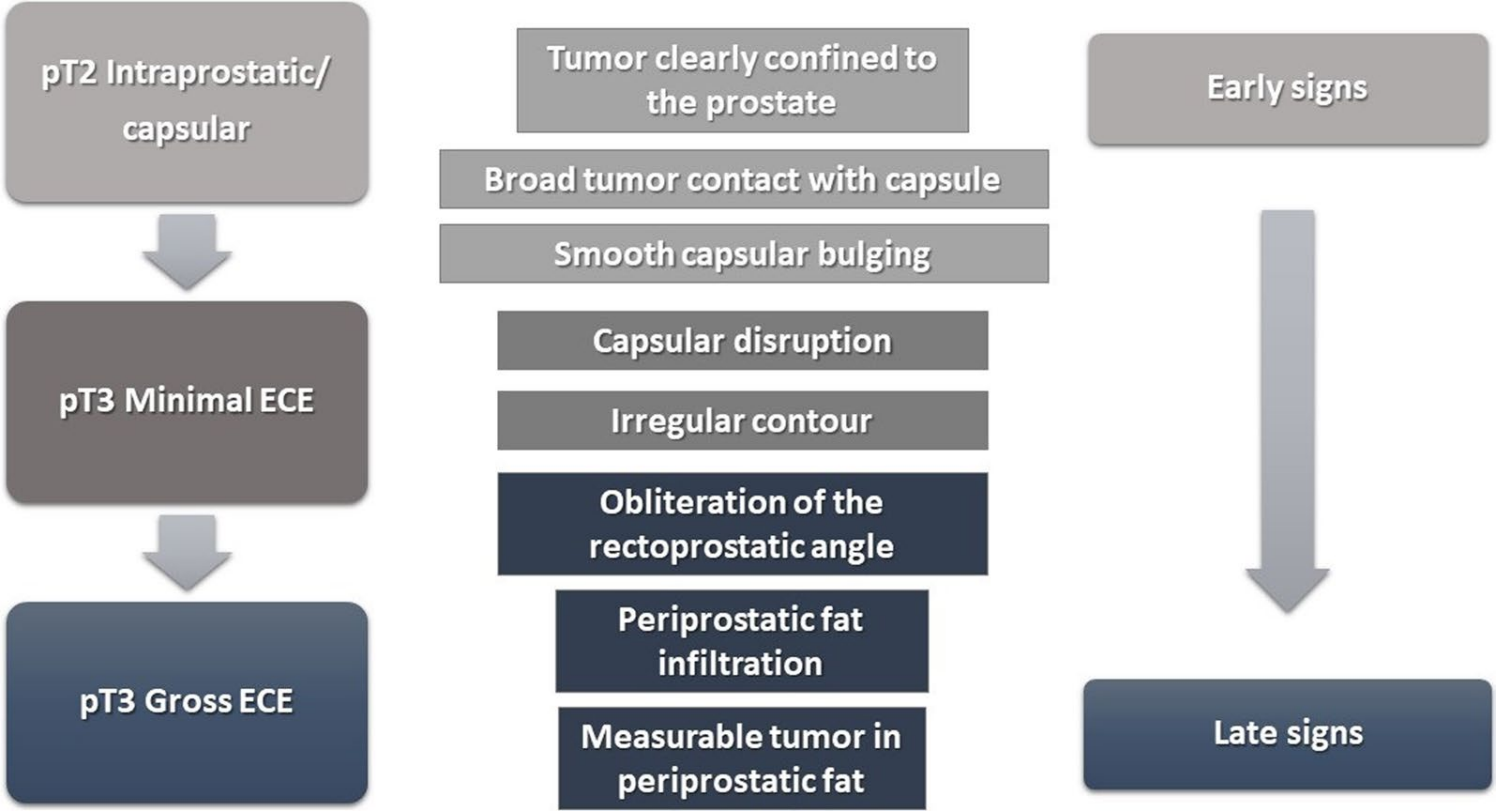
Findings that suggest prostatic cancer extra-capsular extension (ECE) on MRI. These findings can be divided into early and late, as the tumoral invasion progresses from microscopic ECE to perceptible macroscopic tumoral deposits. Reprinted with permission from Flor-de-Lima B, Freire G, Lopes A, et al. (2022) Radiologic-pathologic correlation of prostatic cancer extra-capsular extension (ECE) (C-18805) EPOS™ poster presented at ECR 2022

**Fig. 4:**
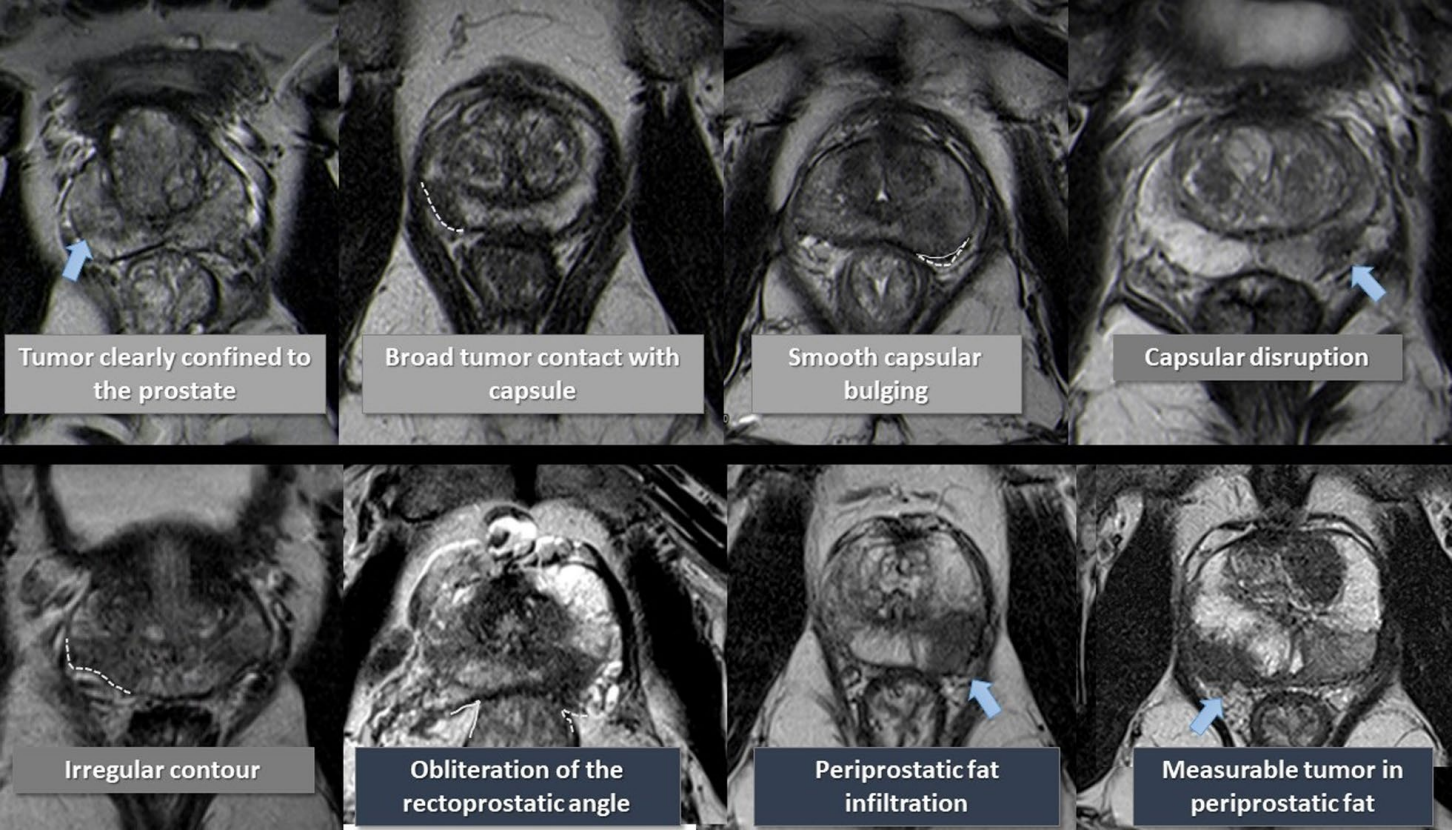
Schematic representation of the findings that advocate prostatic cancer extra-capsular extension (ECE) on axial T2-weighted images of MRI from different patients with prostatic cancer. Reprinted with permission from Flor-de-Lima B, Freire G, Lopes A, et al. (2022) Radiologic-pathologic correlation of prostatic cancer extra-capsular extension (ECE) (C-18805) EPOS™ poster presented at ECR 2022

The detection of ECE on MRI relies on the visualization of subjective findings related to macroscopic extension of the tumors and its mechanical consequences [11]. These findings vary according to the aggressivity of the tumor and the degree of ECE. The features that suggest ECE on MRI can be divided into early and late findings (Figs. 3 and 4). These criteria are based on a chronological concept of cancer growth from truly intraprostatic to extra-prostatic zone, in accordance with recognized histopathologic criteria for ECE.

The early findings of ECE include a wide tumor contact with the prostate capsule, the tumor capsular contact length (TCCL), smooth capsular bulging (smooth projection over the prostatic border), and capsular disruption (defined as a discontinuity in the low signal prostatic margin) [12-14].

As the tumor grows under the capsule, tumoral glands form appreciable deposits that can be seen on MRI as late findings, which comprise: irregular contour of the prostate (loss of the clear interface with the periprostatic fat); obliteration of the rectoprostatic angle (disappearance of the angle formed by the rectum and the prostate gland); periprostatic fat infiltration (replacement of the fat high-signal intensity by low-signal intensity); and, presence of measurable tumor in the periprostatic fat (presence of an obvious tumoral tissue in the periprostatic space).

The late findings have a higher predictive positive value for ECE and its assessment has a higher reproducibility since they are more evident than the early findings [1].

**Fig. 5:**
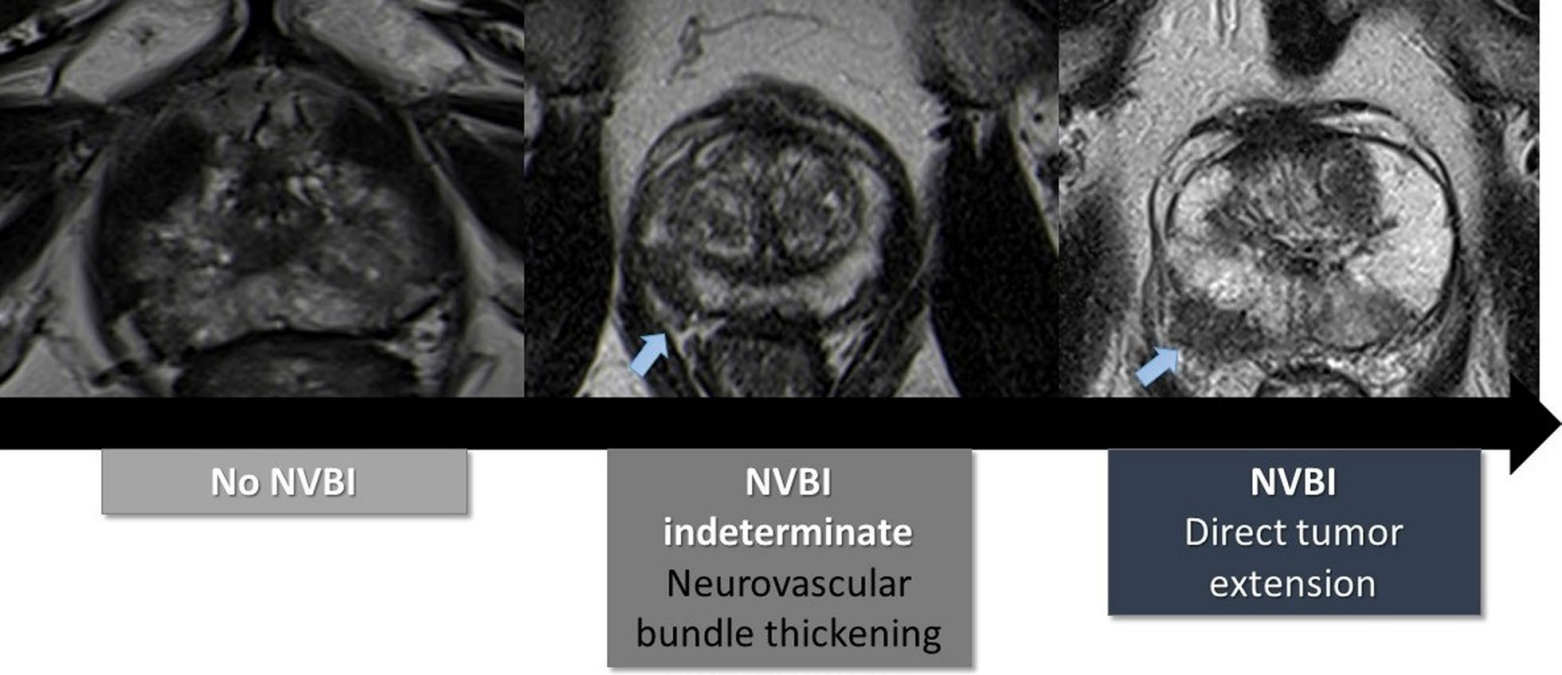
Representation of the findings of neurovascular bundle invasion (NVBI) on T2-weighted images of MRI from different patients with prostatic cancer. Reprinted with permission from Flor-de-Lima B, Freire G, Lopes A, et al. (2022) Radiologic-pathologic correlation of prostatic cancer extra-capsular extension (ECE) (C-18805) EPOS™ poster presented at ECR 2022

In regards to the neurovascular bundle invasion (NVBI), it can be suggested on MRI by the presence of neurovascular bundle asymmetry or by detecting direct tumoral extension (Fig. 5).

## Pathologic findings

### Normal prostate

Microscopically, the normal adult prostate gland is composed by a branching duct-acinar glandular system embedded in a dense fibromuscular stroma. The prostatic parenchyma is divided into three major zones: peripheral, central and transition zone. Within each prostatic zone, the glandular epithelium is generally simple columnar, but there may be patches that are simple cuboidal, squamous, or occasionally pseudostratified. In addition, fibromuscular stroma occupies the anterior surface of the prostate gland anterior to the urethra and is composed of dense irregular connective tissue with a large amount of smooth fibers.

Zonal differences can also be appreciated on pathologic analysis:

- The PZ comprises 70% of the glandular tissue of the prostate, surrounding the central zone and occupying the posterior and lateral parts of the gland. This zone is the most susceptible to inflammation, and it is where most prostatic carcinomas arise from. Normally, the PZ ducts and acini are evenly distributed but are irregular in size and shape [15].
- The TZ surrounds the prostatic urethra; it comprises about 5% of the prostatic glandular tissue. The normal TZ glands are similar to those of the PZ but are embedded in a compact stroma that forms a distinctive boundary with the loose stroma of the PZ [15].
- The CZ contains about 25% of the glandular tissue, with glands being more densely arranged than PZ and TZ glands. The ratio of epithelium to stroma is higher in the CZ than in the rest of the prostate. Also, CZ glands are larger and display intraluminal projections with fibrovascular cores.

From a microscopic and pathologic point of view, the prostate does not have a true capsule, the outer surface of the prostate, also called pseudocapsule, encompasses the exterior stromal edge of the prostate parenchyma, formed by transversely arranged fibromuscular layers of condensed smooth muscle [16]. At the prostate apex, the pseudocapsule is sparser, with more intimate admixture of glandular tissue with sphincteric striated muscle. Its boundaries are also not easily defined at the anterior aspect of the gland and bladder neck regions, creating similar difficulties in assessing the extent of invasion by carcinoma.

### Subtypes of prostate cancer and Gleason score

Prostatic acinar adenocarcinoma accounts for most prostate cancers. Variants of usual acinar adenocarcinoma are, according to the 2016 World Health Organization, the following: atrophic; pseudohyperplastic; microcystic; foamy gland; mucinous (colloid); signet ring-like cell; pleomorphic giant cell and sarcomatoid. The former four variants may seem deceptively benign in histological appearance, such that a misdiagnosis of a benign condition may be made [17]. Ductal adenocarcinoma is the second most common subtype of prostatic carcinoma, frequently located in the periurethral area, often protruding into the urethra. It is considered a more aggressive form of adenocarcinoma, with higher rates of distant metastasis [18].

Intraductal carcinoma is recognized as a new entity in the 2016 WHO classification that encompasses intra-acinar and/or intraductal neoplastic epithelial proliferations, sharing some features of high-grade prostatic intraepithelial neoplasia, with greater architectural and/or cytological atypia. Its recognition is critical as it is commonly associated with high-grade and high-stage prostate carcinoma [19].

There are many other subtypes of prostate cancers, though they are exceedingly rare, given the overwhelming diagnosis of prostatic adenocarcinomas. Some of these other subtypes of cancers include squamous neoplasms, basal cell carcinoma and neuroendocrine tumors. The latter comprises small cell neuroendocrine carcinoma, characterized by scant cytoplasm (high nuclear to cytoplasmic ratio), hyperchromatic nuclei without conspicuous nucleoli, frequent mitosis, fragility, crush artifacts, nuclear molding, rosette-like structures, and geographic necrosis.

**Fig. 6:**
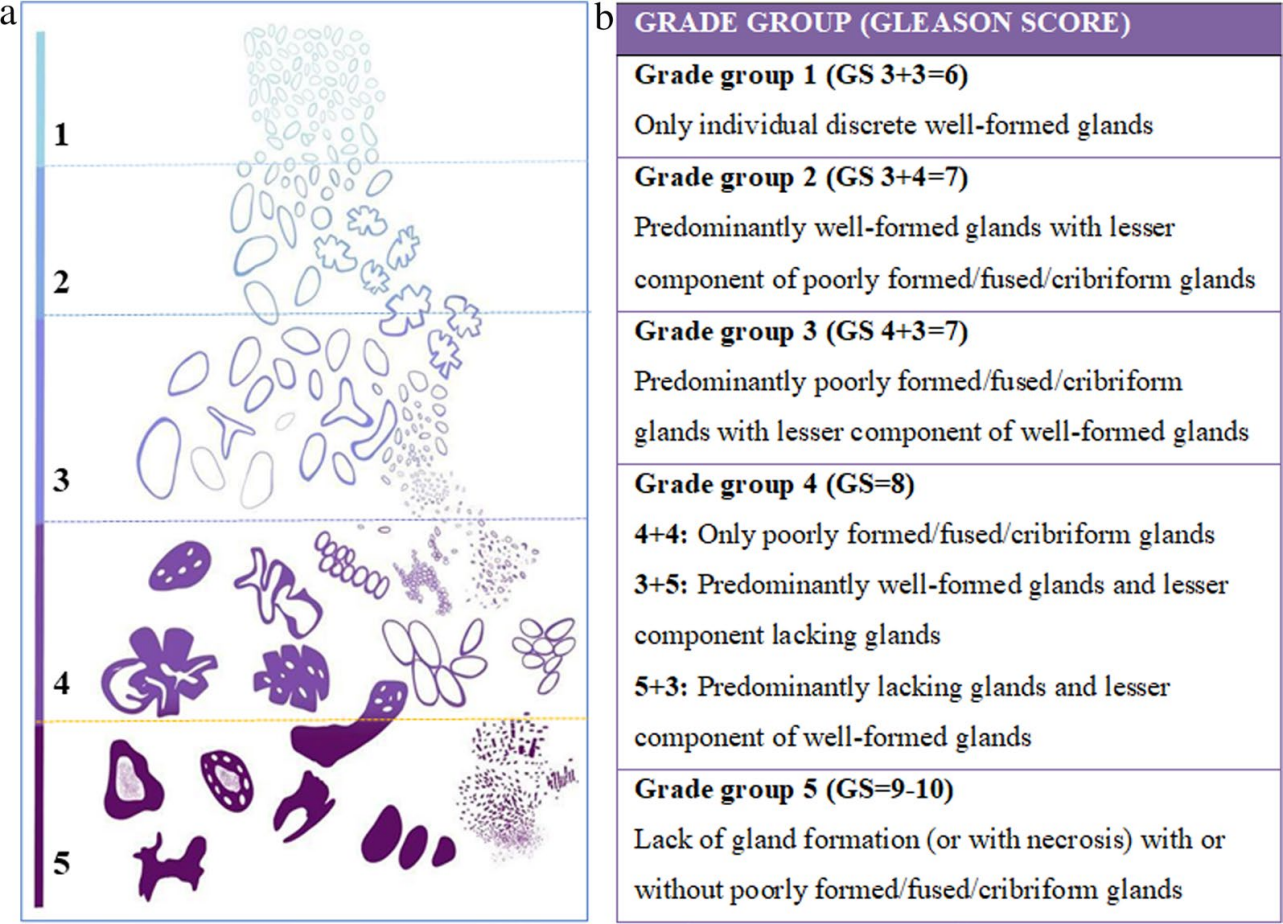
Modified Gleason grading schematic diagram based on 2015 modified ISUP Gleason schematic diagram (**a**). Histologic definition of the International Society of Urological Pathology (ISUP) grading system (**b**)

The Gleason grading system remains the standard approach to histologic grading of adenocarcinoma of the prostate, except for cases showing treatment effects, as seen in the setting of hormonal ablation and radiation therapy [19]. The Gleason score is the sum of the predominant Gleason grade in terms of surface area (primary grade) to the second most prevalent Gleason grade (secondary grade), which are assigned a number from 1 (most differentiated) to 5 (least differentiated). In case there is not a secondary Gleason grade, the primary Gleason grade is doubled to determine a Gleason score. If there is a third pattern of higher grade corresponding to > 5% of the area of the tumor, this number should replace the secondary grade pattern number. Theoretically, the Gleason score ranges from 2 to 10, even though the lowest grade assigned is 6, in which only individual discrete glands are visualized. According to the 2016 World Health Organization, the presence of cribriform, glomeruloid or poorly formed or fused glands is defined as Gleason pattern 4. Whereas Gleason pattern 5 is attributed to sheets of tumor, individual cells, cords, linear arrays, solid nests of cells and presence of comedonecrosis [19]. Gleason scores can also be grouped into five prognostic categories, using ISUP (International Society of Urological Pathology) 5-tier grading system as recommended at the 2014 ISUP  consensus meeting (Fig. 6) [20]. This grading system should be reported as ISUP grade group (GG) with the corresponding Gleason patterns and scores. GG 1 is used for all cases with Gleason scores ≤ 6, including tumors with indolent nature. Gleason 7 can be divided into GG 2 and 3, based on whether Gleason 3 or Gleason 4 predominate, which emphasizes the different prognostic significance between Gleason scores 7 (3 + 4) and 7 (4 + 3). The GG 4 category (4 + 4, 3 + 5 and 5 + 3) consists of tumors that behave in a more aggressive manner than those of GG 3 or lower. GG 5 consists of Gleason scores 9 and 10, being associated with poor prognosis [20].

It should be noted that intraductal carcinoma is not assigned a Gleason grade, though it is often associated with an average Gleason score of 8 and pT3 prostatic adenocarcinoma [21].

### Extracapsular extension-pathology (pECE)

**Fig. 7:**
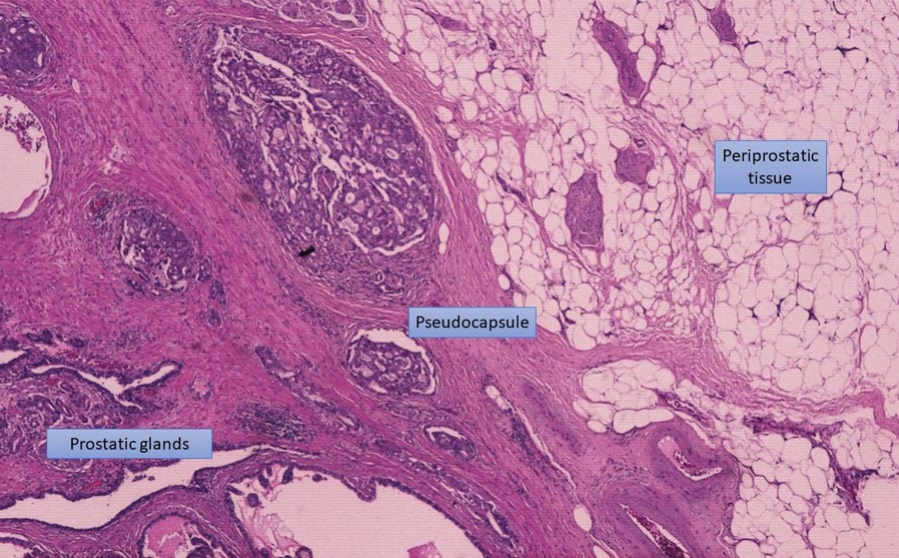
Histological analysis from a prostatectomy specimen shows the pseudocapsule formed by organized layers of condensed smooth muscle. Beyond it, the periprostatic tissue is seen, mainly constituted by adipocytes (Hematoxylin and Eosin (H&E)). Reprinted with permission from Flor-de-Lima B, Freire G, Lopes A, et al. (2022) Radiologic-pathologic correlation of prostatic cancer extra-capsular extension (ECE) (C-18805) EPOS™ poster presented at ECR 2022

At pathology, the evaluation of pECE can be challenging, as the prostate lacks a true capsule and its boundaries are not easily defined, especially at the level of the apex and at the anterior aspect of the gland. The pseudocapsule consists of organized layers of condensed smooth muscle (Fig. 7) that may be intermixed with the prostatic stroma and, thus, may be hard to be correctly delineated [9].

At the apex, the prostatic tissue is mixed with skeletal muscle from the urogenital diaphragm but, if tumoral cells are seen within skeletal muscle, it does not necessarily mean ECE. According to the ISUP consensus [22], there are no definitive criteria to define ECE at the prostatic apex.

Likewise, at the anterior part, the prostatic boundaries are also imprecise, with glandular stroma admixed with fibromuscular tissue from the urogenital diaphragm. However, at that level, ECE is defined by the presence of tumoral glands outside the contour of the normal prostatic tissue [22].

The diagnosis of pECE implies the presence of one of the following criteria [23]:

- Neoplastic cells are seen in the periprostatic fat;
- If tumoral glands are seen surrounding the nerves in the neurovascular bundles;
- If there is a tumoral extension beyond the periphery of the prostate.

The degree of ECE can be then divided into focal or extensive. Focal extension is present if the tumoral glands do not occupy more than one high-power field on no more than two separate histopathological sections, whereas it is defined as extensive if the extension is superior to the stated [24].

The evaluation of EPE on pathology remains a challenge depending on the evaluation method and the interpretation of the pathologist [25], which might have a negative impact on MRI accuracy when its used a gold standard.

## Radiologic-pathologic correlation and pitfalls

**Fig. 8:**
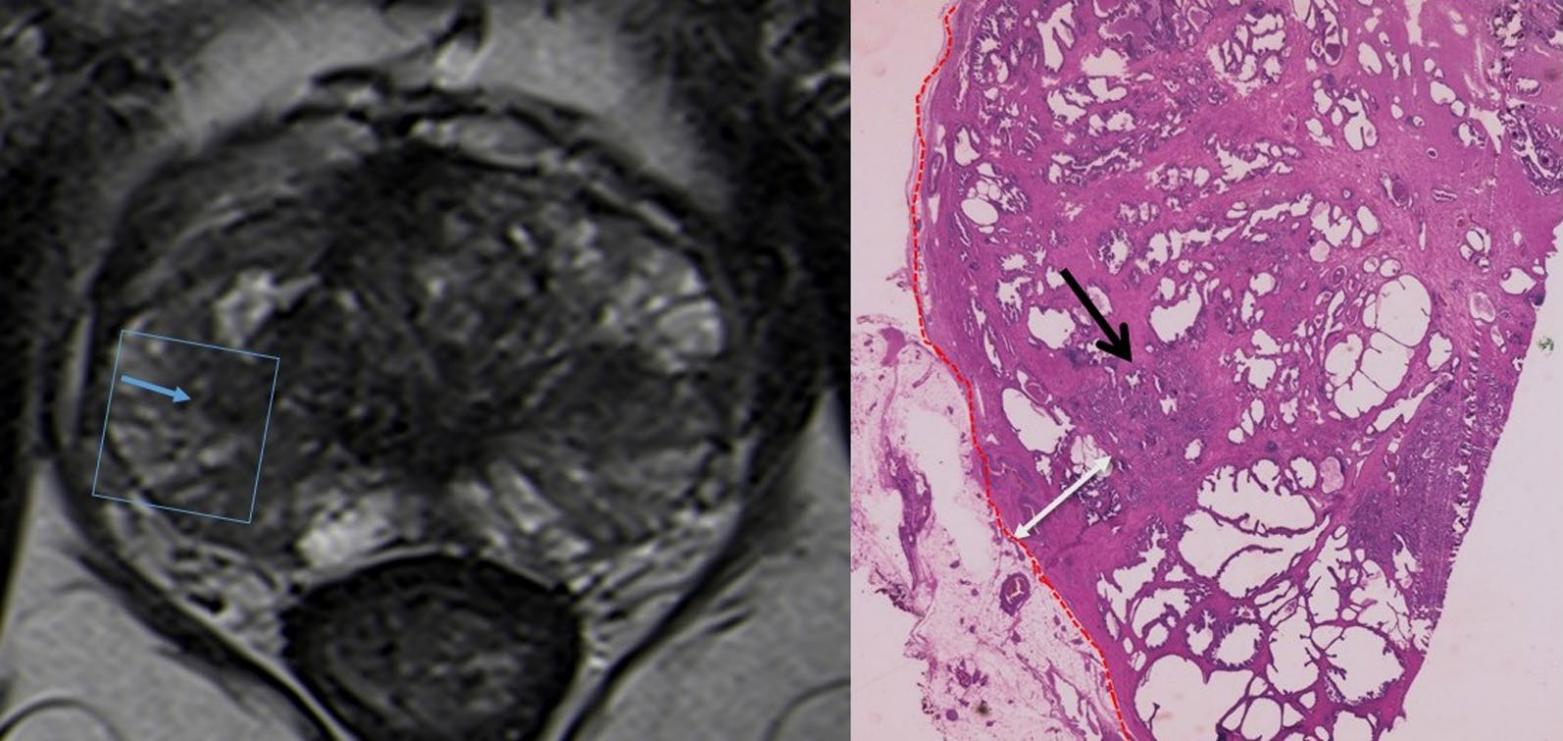
T2-WI MR image shows a low signal intensity right lesion (blue arrow) that shows no capsular contact or other signs of suspicious extracapsular extension. The correspondent histological analysis confirms the tumor (dark arrow) with no contact with the capsule (red line). (Hematoxylin and Eosin (H&E)). Reprinted with permission from Flor-de-Lima B, Freire G, Lopes A, et al. (2022) Radiologic-pathologic correlation of prostatic cancer extra-capsular extension (ECE) (C -18,805) EPOS™ poster presented at ECR 2022

**Fig. 9:**
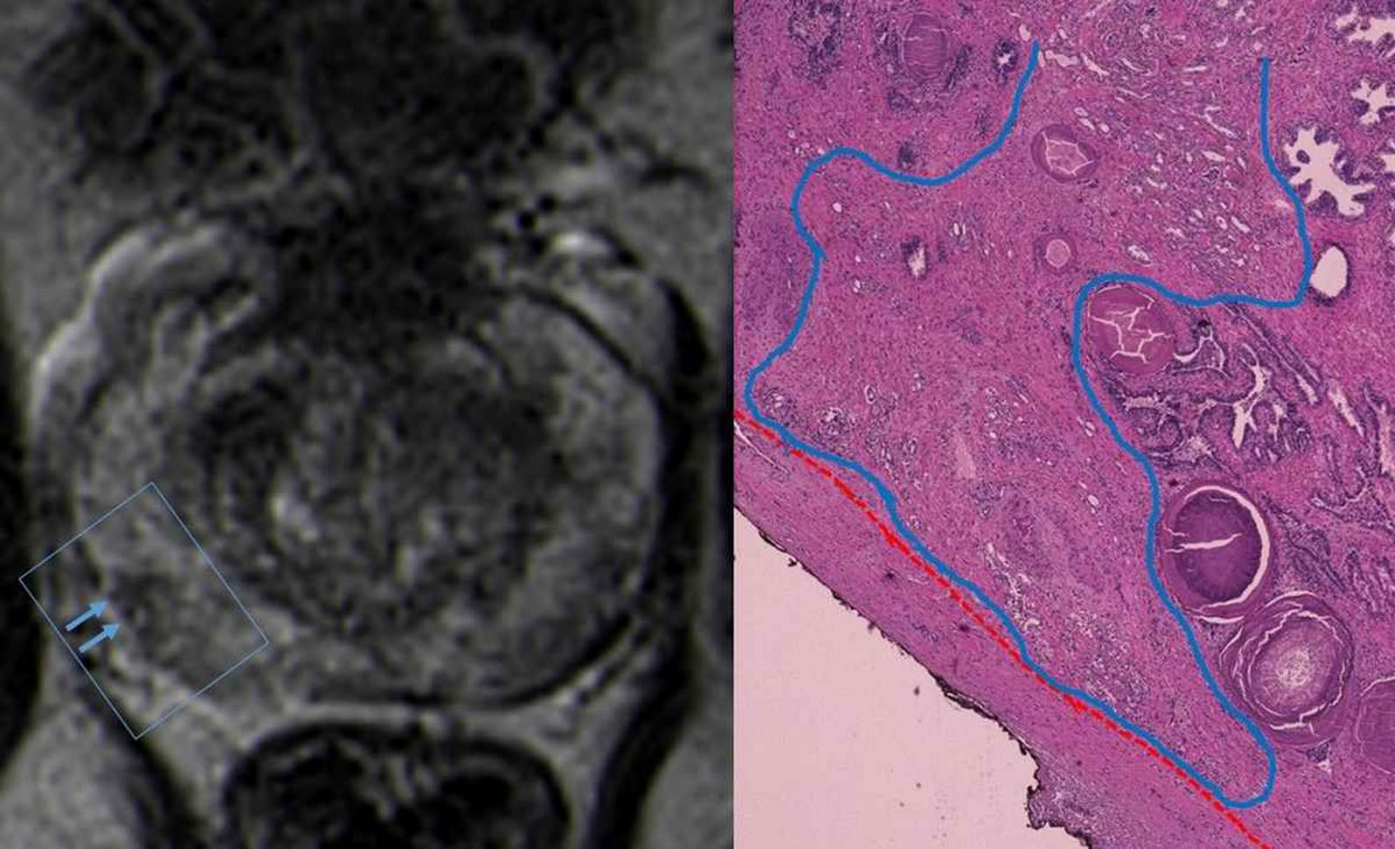
T2-WI MR image shows a low signal intensity right peripheral lesion with narrow contact with the capsule (blue arrows), with no bulging, only irregularity of the capsular contour. The correspondent histological analysis reveals narrow tumoral capsular contact (tumor margin lined in blue), measuring 2.6 mm in extension. The capsule is lined in red. (Hematoxylin and Eosin (H&E)). Reprinted with permission from Flor-de-Lima B, Freire G, Lopes A, et al. (2022) Radiologic-pathologic correlation of prostatic cancer extra-capsular extension (ECE) (C-18805) EPOS™ poster presented at ECR 2022

**Fig. 10:**
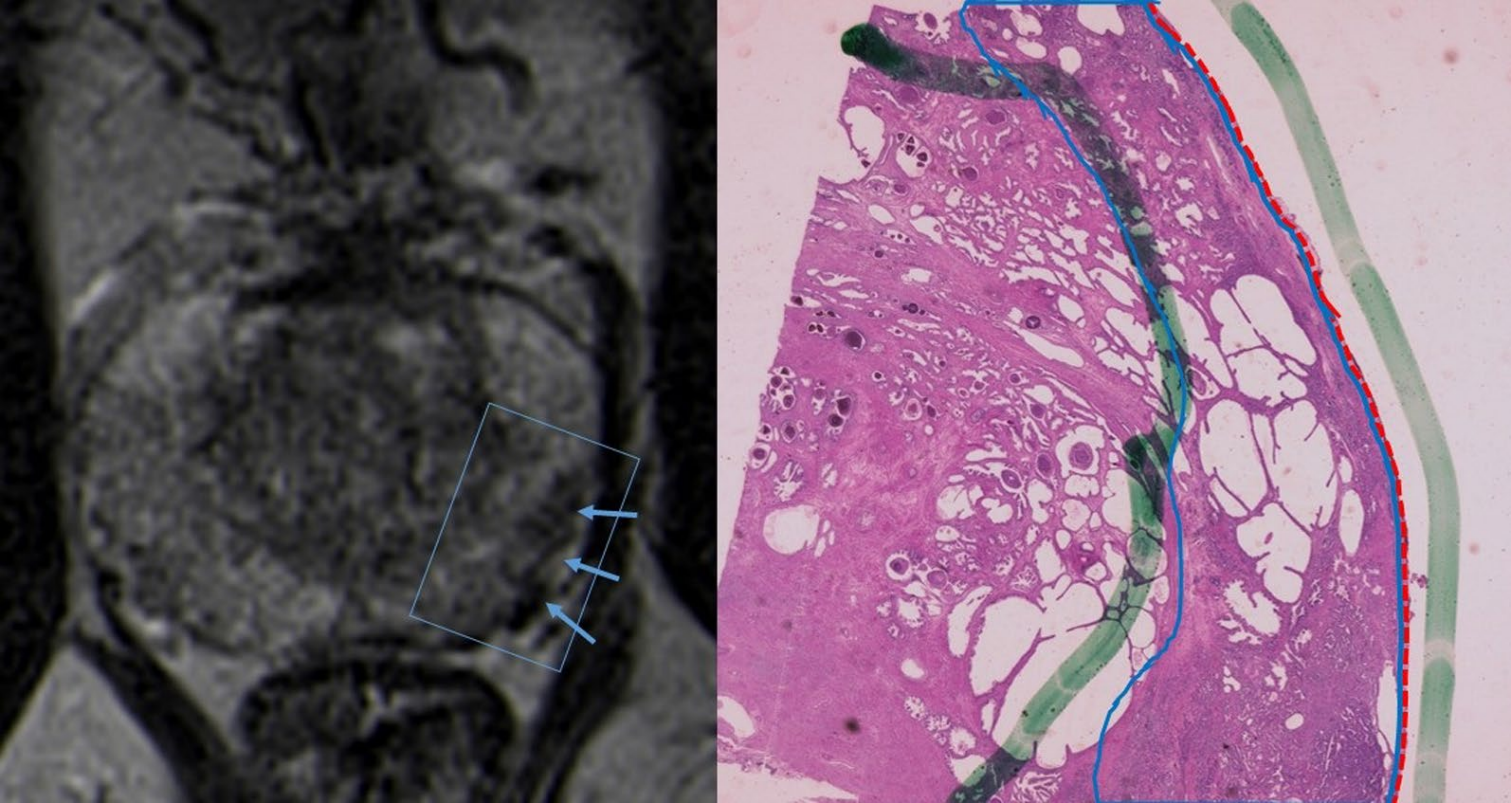
T2-WI MR image shows a low signal intensity left peripheral lesion with broad contact with the capsule (blue arrows) but with no budging, only irregularity of the capsular contour. The correspondent histological analysis reveals the tumor (lined in blue) contacting the prostatic capsule (red line). This contact has 26 mm in extension, but there is no extracapsular extension (Hematoxylin and Eosin (H&E)). Reprinted with permission from Flor-de-Lima B, Freire G, Lopes A, et al. (2022) Radiologic-pathologic correlation of prostatic cancer extra-capsular extension (ECE) (C-18805) EPOS™ poster presented at ECR 2022

**Fig. 11:**
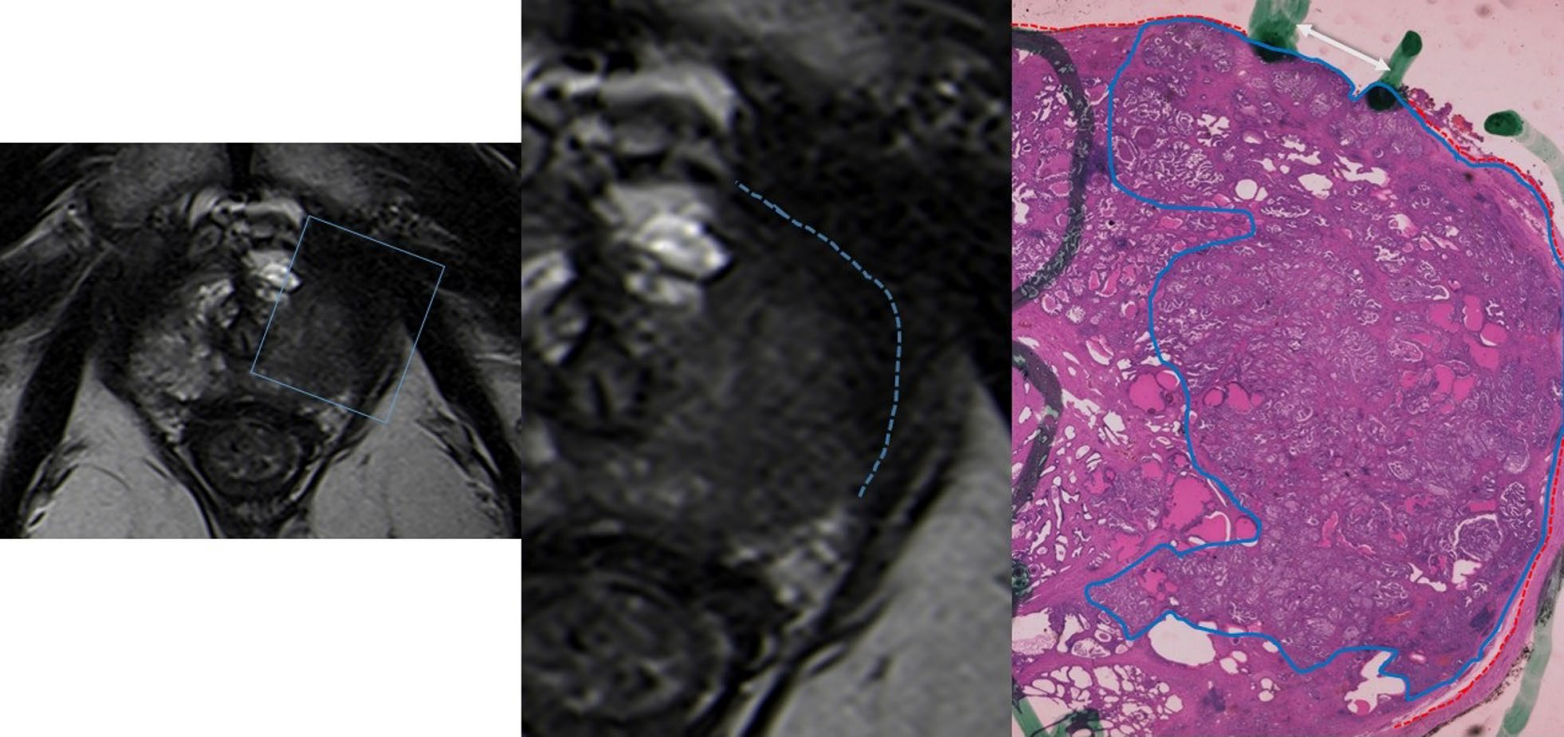
T2-WI MR images with zoom out and in reveal a low signal intensity left lesion that shows broad contact with the capsule (dashed blue line). The correspondent histological analysis reveals tumor (blue line) with extensive tumoral capsular contact of more than 38 mm (dashed red line). There are also positive surgical margins located between the two green lines and marked with a white arrow (Hematoxylin and Eosin (H&E)). Reprinted with permission from Flor-de-Lima B, Freire G, Lopes A, et al. (2022) Radiologic-pathologic correlation of prostatic cancer extra-capsular extension (ECE) (C-18805) EPOS™ poster presented at ECR 2022

**Fig. 12:**
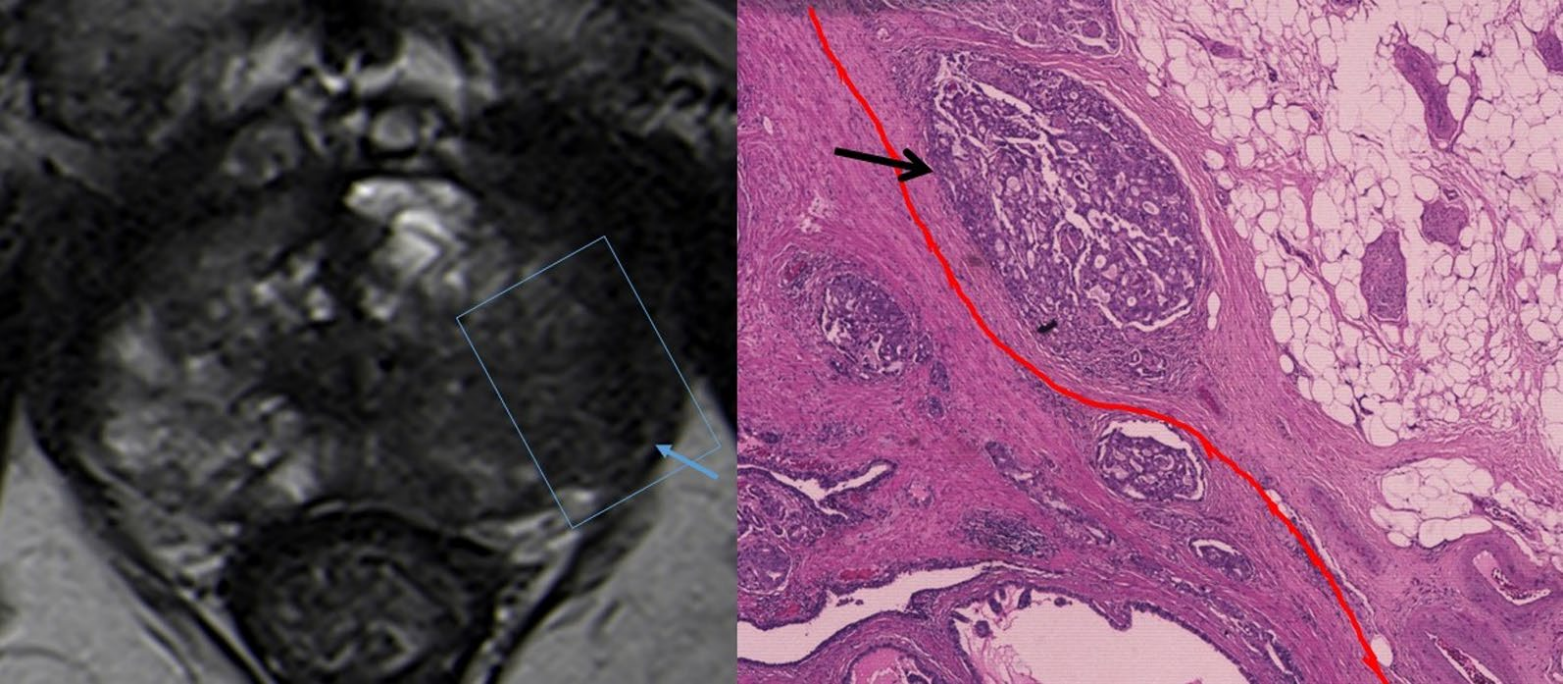
T2-WI MR image reveals a low-signal intensity peripheral lesion that shows broad contact with the capsule, with bulging and ECE (blue arrow). The correspondent histological analysis reveals small tumoral foci (one marked with an arrow) beyond the prostatic capsule (red line), corresponding to extraprostatic extension (Hematoxylin and Eosin (H&E)). Reprinted with permission from Flor-de-Lima B, Freire G, Lopes A, et al. (2022) Radiologic-pathologic correlation of prostatic cancer extra-capsular extension (ECE) (C-18805) EPOS™ poster presented at ECR 2022

MRI has improved significantly the ability to depict the intraprostatic location of clinically significant cancers (> 0.5 mL, Gleason score > 6) [26]. If this MRI observed lesion does not abut the prostatic capsule, ECE is very unlikely to occur (Fig. 8). The likelihood of ECE will increase with increasing tumor-capsular contact length. As the tumor grows along the prostate capsule, the malignant cells tend to contact, invade and disrupt prostate capsule giving the early ECE findings on MRI [1]. These early findings of ECE include a wide tumor contact with the prostate capsule; smooth capsular bulging (smooth projection over the prostatic border); and capsular disruption, defined as a discontinuity in the low signal prostatic margin (Figs. 9, 10, 11, 12).

**Fig. 13:**
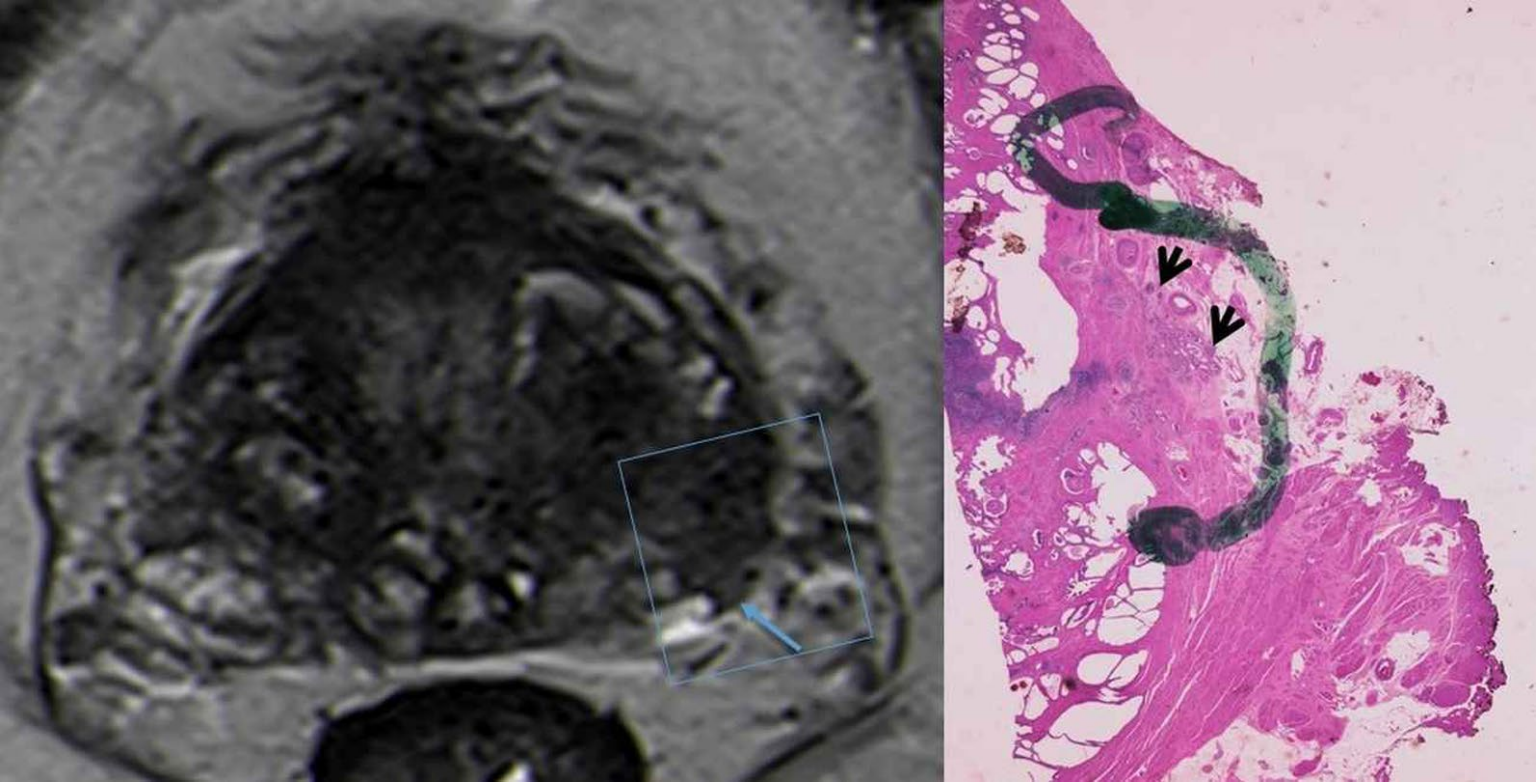
T2-WI MR image reveals a low signal intensity peripheral lesion, at the base, that contacts the prostate capsule (broad contact) associated with irregular prostatic contour and periprostatic fat infiltration (blue arrow). The correspondent histological analysis reveals tumoral foci (arrows) beyond the expected prostatic capsule, corresponding to extraprostatic extension (Hematoxylin and Eosin (H&E)). Reprinted with permission from Flor-de-Lima B, Freire G, Lopes A, et al. (2022) Radiologic-pathologic correlation of prostatic cancer extra-capsular extension (ECE) (C-18805) EPOS™ poster presented at ECR 2022

After these early or “microscopic” stages of ECE, malignant cells grow further away from the capsule and clump together into late or “macroscopic” deposits. The prostatic contour becomes irregular, unsharped and later on, the tumor invades the periprostatic with replacement of the fat high-signal intensity by low-signal intensity on T2 and the presence of an obvious tumoral tissue in the periprostatic space (Fig. 13). Table 1 represents the clinical, MRI and pathological correlations of the presented patients.

**Table 1:** Clinical information of the patients analyzed in the figures comparing the GS, MRI stage and the PI-RADS score of the PCa and the rad-path correlation, respectively

Participant ID	MRI stage	Gleason score (GS)	PI-RADSv2.1	Rad-path correlation
1	iT2	6 (3 + 3)	PI-RADS 3	pT2. Figure 7
1 (index lesion)	iT3a	7 (3 + 4)	PI-RADS 3	pT3a. Figure 12
2 (index lesion)	iT2	7 (3 + 4)	PI-RADS 4	pT2. Figure 9
2	iT2	6 (3 + 4)	PI-RADS 3	pT2. Figure 8
3	iT3a	7 (4 + 3)	PI-RADS 5	pT3a. Figures 10, 11

As MRI macroscopically visualizes events that happen on a microscopic scale, some stages along the evolutionary timeline are difficult to depict, and its visualization depends on the MRI protocols and the conditions of the MRI examination. The early signs of ECE could also be seen in non-ECE involvement cases, and the microscopic prediction in MRI imaging is not easy. Conversely, the presence on ECE measurable on MRI is related almost 100% of presence of pECE on pathology [1, 14, 27, 28].

The late signs of ECE tend to be greater agreement between readers than the early signs, which may not be very reproducible [1, 14, 27, 28].

Some MRI-based methods aim to reduce the interpretative subjectivity of readers to diagnose ECE on MRI. One method is the extraprostatic extension (EPE) grade which combines MRI semantic features and TCCL > 1.5 cm, into three grades, from Grade 1 (24%) to Grade 3 (66%) risk for the presence of pECE [14]. EPE includes the ECE and could also include SVI. The European Society Urogenital Radiology ESUR scoring system is another method that uses qualitative descriptors in to 5 grades to predict the probability of ECE, similar to the PI-RADS score used for predicting tumor aggressiveness [4, 14, 29]. A quantitative measurement of TCCL has also been proposed as an individual biomarker to predict ECE. The cut-off for TCCL varies between 10 and 20 mm in different studies [30]. Additionally, a predictive model was published that combines clinical, MRI features and Gleason score (GS) from prostate biopsy, and the author found that the TCCL, measurable ECE, capsular disruption and GS > (3 + 4) were the significant predictors to ECE [3].

The main anatomical challenge to be aware of when interpreting MRI findings is the periprostatic venous plexus and NVB. These structures are very proximal to the posterior lateral contour of the prostate capsule and they can create difficulties in distinguishing between potential lesions and the normal anatomy. They exhibit round appearance with low signal intensity on T2WI, mimicking a prostate lesion. In addition, they may cause anatomic distortion of the prostate boundaries, which can also be misinterpreted as a mass lesion within or in periprostatic space. The high spatial resolution T2WI can be used to identify the typical bilateral and symmetric, tubular morphology of the NVB coursing along the lateral margin of the peripheral zone [4, 31].

There are other imaging pitfalls that could simulate a T3 disease in prostate which may include anatomical variation as of the shape of the prostate apex, BPH nodules that bulge along root of seminal vesicles, and granulomatosis prostatitis that can infiltrate the peri-prostatic fat [31].

This Rad/Path correlation suggests that the goal of a staging MRI should be to predict the likelihood of EPE on a scale from low (early signs) to high (late signs) as been used by other authors and added other clinical information as preoperative PSA, clinical stage and Gleason score on prostate biopsy in order to predict the early microscopic pathologic stages of PCa [14, 27, 28].

## Conclusion

The MRI is the imaging method to stage PCa before surgery with a good anatomic correlation of prostate gland and periprostatic space with pathology. The MRI early features of pECE are not so consensual and predictive as MRI late features of pECE.

## Data Availability

All data and materials presented were from our hospital and daily practice.

## Abbreviations

CZ: Central zone
ECE: Extracapsular Extension
EPE: Extraprostatic Extension
GG: Grade Group
H&E: Hematoxylin and Eosin
MRI: Magnetic Resonance Imaging
NBV: Neurovascular bundle
PCa: Prostatic Cancer
PZ: Peripheral zone
TZ: Transition zone
